# Lamotrigine as monotherapy in clinical practice: efficacy of various dosages in epilepsy

**DOI:** 10.1002/brb3.419

**Published:** 2016-02-10

**Authors:** Anton Warshavsky, Anda Eilam, Ronit Gilad

**Affiliations:** ^1^ Neurology DepartmentE. Wolfson Medical CenterHolonIsrael; ^2^ Sackler Faculty of MedicineTel Aviv UniversityTel AvivIsrael; ^3^ Neurology DepartmentKaplan Medical Center Hebrew University of JerusalemRehovotIsrael

**Keywords:** Dosage, efficacy, epilepsy, lamotrigine, monotherapy, seizures

## Abstract

**Objectives/Aims:**

The study was designed to evaluate the optimal dosage of lamotrigine, as monotherapy, in the treatment of adults suffering from complex partial seizures with or without secondary generalization in everyday clinical practice.

**Materials and Methods:**

The ones used in this study was the collection of the data of all adult patients treated with lamotrigine, retrospectively. The dosage and efficacy of treatment were evaluated along with side effects and retention rate.

**Results:**

They showed that, out of 188 patients, 77% continued lamotrigine treatment; the mean effective dose was 250 mg or higher of lamotrigine, and the results more pronounced in older patients (age above 30 years) and those with a longer disease duration (5 years and more).

**Conclusion:**

It may be appropriate to reach a daily lamotrigine dose above 250 mg in adult patients suffering from epilepsy for more than 5 years using lamotrigine as monotherapy.

## Introduction

Epilepsy is a neurological disorder characterized by recurrent unprovoked seizures, resulting from excessive neuronal activity in the brain. The lifetime prevalence of epilepsy is estimated to be 1.2–2.9% (Elliot et al. 2008; Syed and Sugatovic 2010). In general, epilepsy is related to an increased risk of injury and mortality. A cornerstone of epilepsy management is the use of AEDs (antiepileptic drugs). However, suboptimal use of AEDs is common in clinical settings and can be a reason for breakthrough seizures. Monotherapy is preferred as it is associated with better compliance, fewer side effects, less drug interaction and less teratogenicity.

Lamotrigine (Lamictal) (Manufacturer‐GSK, Novolog) is one of the newer antiepileptic drugs used around the world with FDA approval since 1994. It was first used as add‐on therapy in partial and generalized epilepsy. Lamotrigine is an effective drug considered to be a broad‐spectrum AED with the chemical name of 35‐diamino‐6‐1,2,4‐ triazine. It is available in Israel as a twice daily intermediate‐release formula. Lamotrigine voltage‐sensitive sodium channels primary action, associated with a reduction in glutamate release, produces stabilization of neural membranes by inhibiting excitatory neurotransmitter release, with an additional activity on voltage calcium channels (Nissinen et al. 2004). Target dosing of lamotrigine ranges from 50 to 500 mg/day, depending on concomitant medications. As yet, the optimal dose of lamotrigine, when given as a monotherapy, has still to be determined. In a few pivotal studies, the maximum recommended daily dosage was 200 mg (Matsuo et al. 1993; Glauser et al. 2013). However, clinical experience has shown that this daily dosage is not sufficient in many cases to achieve seizure freedom. Furthermore, doses of up to 500 mg do not increase significantly due to drug toxicity. Therefore, this study was designed to evaluate the optimal dosage use of lamotrigine in the treatment of adults using it as monotherapy for CPS seizures with or without secondary generalization only in everyday clinical practice.

## Materials and Methods

This study was approved by the hospital's medical ethics committee. The data of all adult patients treated with lamotrigine as monotherapy was collected retrospectively from two epilepsy tertiary medical centers using the computer medical database in Israel during the years 2011–2014. Parameters in the database included patient characteristics (age, sex, and type of seizure), duration of epilepsy, frequency of seizures, duration of treatment, dosage of lamotrigine, efficacy, reported side effects, and reasons for discontinuation of lamotrigine. The type of seizure was classified using the ILAE (International League Against Epilepsy) classification (Fisher et al. 2005). Positive efficacy of treatment was defined as being seizure free. During the study, determination of plasma lamotrigine levels was not available in our country.

This study was a longitudinal and observational one. Included in the survey were patients undergoing medical observation in two medical centers. The patients visited the outpatient clinics every three months for evaluation of their seizure diaries and lamotrigine (LTG) dose usage. The protocol for treatment adopted by the two medical centers included monotherapy of LTG, increasing the dosage with 25 mg every week bid up until a dosage level of 100 mg bid. If more seizures were observed, the dosage was increased boosted up until a maximum dose of 400 mg bid was reached. If more seizures occurred needing above this dosage level, the drug was changed to another antiepileptic medication.

Inclusion criteria incorporated all adult patients, 18 years and older, suffering from epilepsy and being treated with lamotrigine as monotherapy. The type of epilepsy researched was CPS only, with or without generalization, in order to extend the bias of treatment efficacy according to type of seizure. Exclusion criteria included patients under the age of 18 years, ones treated with lamotrigine as an add‐on therapy and ones with idiopathic generalized tonic–clonic seizures. If there was missing data about number of seizures or efficacy of lamotrigine treatment, information was coalesced by a follow‐up phone call in response to questionnaire information. Statistical analysis was performed using SPSS 14.0 (SPSS Inc., Chicago, IL) for Windows. The variability was tested by ANOVA test. Other data were evaluated using an independent *t* test and Pearson χ^2^ test.

## Results

A total of 188 patients treated with lamotrigine as monotherapy were included in the study. To outline the efficacy results, the patients were subdivided into three groups: Group 1 included 68 patients treated with an efficient dosage of lamotrigine up to 200 mg/day; Group 2 included 78 patients treated with an efficient dosage of lamotrigine above 250 mg/day; and Group 3 included 42 patients who were in lamotrigine failure because of loss of efficacy, defined as increase or no change in seizure frequency, or because of side effects (Table 1).

**Table 1:** Characteristics of the patient groups

Parameters	LTG up to 200 mg/day	LTG > 250 mg/day	LTG failure	Total	*P*
Number	68	78	42	188	
Mean age (years)	32.0 ± 16.5	44.0 ± 21.6	39.0 ± 17.0	38.7 ± 19.4	0.036
Duration of disease (years)	6.1 ± 5.5	8.7 ± 6.2	11.6 ± 7.6	8.4 ± 6.6	0.009
Sex M/F	26/42	32/46	8/34		0.214
(%)	(8/62)	(41/59)	(19/81)		
Type of seizures: Focal/Focal + Sec. Generalized	44	54	24	122	
(%)	36.1	44.3	19.7		
GTCS	24	22	16	62	0.678
(%)	38.7	35.5	25.8		

The patients, who needed a higher dosage of lamotrigine (>250 mg/day) to control their seizures, were older, respective to the subgroup, who were seizure free or under a dosage of lamotrigine 200 mg/day. This difference between groups was statistically significant (*P* = 0.036) (Table 2).

**Table 2:** Correlation between dosage of LTG and age

Age (years)	LTG up to 200 mg/day	LTG > 250 mg/day	LTG failure	Total	*P*
18–30 (No.)	46	24	16	86	
(%)	53.3	27.9	18.6		
31–50 (No.)	10	26	14	50	
(%)	20	52	28		
>51 (No.)	12	28	12	52	
(%)	21.3	53.8	23.1		<0.029

These results were found in the patient group of ages 30–50 or above 50 years, who responded to a higher dose of lamotrigine, having twice the efficacy respective to patients below the age of 30 years. A correlation between duration of seizures and dosage of lamotrigine was found to be statistically significant, meaning that patients with a longer duration of disease (above 5 years) needed a higher dosage of lamotrigine to control their seizures (*P* = 0.009) (Table 3). There was no correlation found between sex and lamotrigine dosage (*P* = 0.21), nor between type of seizure and dosage of lamotrigine (*P* = 0.678).

**Table 3:** Correlation between duration of disease and dosage of LTG

Duration of disease	LTG up to 200 mg/day	LTG > 250 mg/day	LTG failure	Total	*P*
Up to 5 years	42	30	12	84	
(%)	50	35.7	28.6		
>5 years	26	48	30	104	
(%)	25	46.2	28.8		<0.033

To show the correlation between patient age and treatment efficacy, the total 188 patients were further divided into three subgroups according to age (up to 30 years, 30–50 years, and above 50 years). In the younger group of patients (up to 30 years), 53.3% of these responded to the lamotrigine dosage of up to 200 mg/day, and in the older group, only 20.0% needed 200 mg of lamotrigine. However, a further 50% of patients needed even a higher dosage (Table 2), so that the differences between the groups were statistically significant (*P* ≤ 0.029).

Regarding consideration of disease duration, the whole group of patients was divided into another two subgroups – patients (84) having a disease duration up to 5 years and patients (104) having a disease duration above 5 years. In those with less than 5 years, 50% needed only 200 mg/day of lamotrigine to control seizures; whereas in the group of patients >5 years of disease duration, only 25% of patients responded to 200 mg/day of lamotrigine. This difference was statistically significant (*P* < 0.033) (Table 3).

Out of the total 188 patients, 42 (22%) discontinued the lamotrigine treatment – 6 (3.1%) had to stop because of side effects (one for severe leukopenia, three for ataxia and headaches, and two for skin eruptions). The remaining 36 (19%) patients discontinued LGT treatment because of nonefficacy, part of them switching treatment to another antiepileptic drug and the others incorporating another add‐on antiepileptic drug. In total, 30 (16%) patients suffered from varying degrees of side effects, causing only six to discontinue treatment and 24 did not – theirs being mild to moderate. The difference between groups was not significant (Table 4).

**Table 4:** Side effects related to dosage

	Lamotrigine up to 200 mg/day	Lamotrigine > 250 mg/day	Lamotrigine failure	*P*
Side effects	14	16	36	
(%)	20.5	20.5	19	0.22

The most frequent side effects were headache [9], vertigo [4], nausea and vomiting [6], and abnormal liver function test [5].

## Discussion

The suggested lamotrigine dosage for monotherapy treatment is 200 mg/day. However, during the period of clinical experience, it has been suggested that this dosage is insufficient in clinical daily practice; so the rationale for this study was that escalation of the lamotrigine dosage from the start may be worthwhile for better efficacy. And indeed, according to our results, there is a statistical connection between lamotrigine dosage efficacy respective to age and duration of disease. In that, patients, with a longer duration of seizures and age higher than 30 years, need from the start of lamotrigine treatment, a higher dosage above 250 mg/day and should not have to wait to undergo breakthrough seizures in order to have an increased dosage.

The mean dosage of monotherapy lamotrigine used today in clinical practice is 200 mg/day. In a study by Giorgi et al. (2001) regarding a meta‐analysis of elderly patients treated with lamotrigine as monotherapy, a mean of 100 mg/day of lamotrigine (range 75–500 mg/day) was established. In our study, the mean dosage of real‐life clinical practice was 290 mg/day, and the mean dosage of lamotrigine failure was 370 mg/day. Out of 188 lamotrigine‐treated patients, only 42 (22%) discontinued the drug and 68% of patients continued treatment with lamotrigine for at least 1 year of follow‐up with good efficacy and minor side effects – suggesting a good tolerability for this drug. Our results are similar to those of Cielewska et al.'s study (2001), which had good response to treatment in 67% of patients and only 21% having side effects, with younger participants. Also, similar to our results, in a study published by Knoester et al. (2005), only 25% of patients discontinued taking lamotrigine after the first year, out of 3,598 patients.

Out of 188 patients, 144 continued LTG treatment, a RR (retention rate) of 78%. This is a relatively high percentage respective to other AED studies and similar to ones as reported by Chung et al. (2007), showing a RR of 74% for lamotrigine. Additionally, a study reported by Boostma et al. (2008) showed similar results to our study with a RR of 74.9% in lamotrigine‐treated patients. The patients in this study discontinued treatment because of lack of efficacy, but this group of patients was also treated with a lower level of drug dosage respective to the group which continued the drug. In our study, a higher dosage of lamotrigine was decided upon especially because of the participation of older patients and those who had suffered from longer periods of illness.

One theory to explain the efficacy of a higher dosage of lamotrigine in long‐standing epilepsy could be a neuroprotective role, but this was not substantially demonstrated. In the work of Nissinen et al. (2004), an explanation of a higher dosage needed in older age could be the changes in the metabolism and resulting drug to drug interaction; but in our study, the higher dosage of lamotrigine was also needed in the middle‐aged group (30–50 years), so this theory is not the compete explanation.

There were some limitations in our study: firstly, the fact that it was a retrospective one and, secondly, that it was impossible at that time to measure lamotrigine serum levels because this testing was not available in Israel. However, it has to be taken into consideration that the therapeutic level range of lamotrigine is very broad and not always very effective in optimizing the LTG therapeutic serum levels.

In conclusion, the results of the study done in a daily clinical practice setting suggest that age and duration of disease are the major factors that influence the dosage efficacy of lamotrigine, as monotherapy in adult epileptic patients. Therefore, it is recommended that in these subgroups of adult epileptic patients treated with lamotrigine as monotherapy, it is worthwhile initiating treatment starting with doses of 250 mg/day or higher, having no evidence for increased side effects.

## Conflict of Interest

None declared.
